# A new data driven method for summarising multiple cause of death data

**DOI:** 10.1186/s12874-023-01901-z

**Published:** 2023-04-05

**Authors:** Annette Dobson, Paul McElwee, Mohammad Reza Baneshi, James Eynstone-Hinkins, Lauren Moran, Michael Waller

**Affiliations:** 1 University of Queensland, School of Public Health, Brisbane, QLD Australia; 2 Australian Bureau of Statistics, Brisbane, Australia

**Keywords:** Death rates, Multiple causes of death, Multimorbidity, Data-driven method

## Abstract

**Background:**

National mortality statistics are based on a single underlying cause of death. This practice does not adequately represent the impact of the range of conditions experienced in an ageing population in which multimorbidity is common.

**Methods:**

We propose a new method for weighting the percentages of deaths attributed to different causes that takes account of the patterns of associations among underlying and contributing causes of death. It is driven by the data and unlike previously proposed methods does not rely on arbitrary choices of weights which can over-emphasise the contribution of some causes of death. The method is illustrated using Australian mortality data for people aged 60 years or more.

**Results:**

Compared to the usual method based only on the underlying cause of death the new method attributes higher percentages of deaths to conditions like diabetes and dementia that are frequently mentioned as contributing causes of death, rather than underlying causes, and lower percentages to conditions to which they are closely related such as ischaemic heart disease and cerebrovascular disease. For some causes, notably cancers, which are usually recorded as underlying causes with few if any contributing causes the new method produces similar percentages to the usual method. These different patterns among groups of related conditions are not apparent if arbitrary weights are used.

**Conclusion:**

The new method could be used by national statistical agencies to produce additional mortality tables to complement the current tables based only on underlying causes of death.

## Background

Summary data on the causes of death are used to allocate health system resources for prevention and treatment of disease and to monitor disease trends over time. National mortality statistics are typically based on single causes of death. Until the mid twentieth century this was appropriate while infectious diseases were the primary causes of most deaths. However, as living standards, effective treatments, and disease prevention and control measures have improved, longevity has increased and as more of the world’s population is becoming older, multi-morbidity (suffering two or more chronic conditions simultaneously) is becoming increasingly common. For older people, the single cause of death is less realistic for describing disease burden [1, 2]. As Désesquelles et al. note “At old ages, death is indeed often the final stage of a long morbid process involving several conditions” [3].

There is a World Health Organization (WHO) framework for recording causes of death [4]. For each death a doctor should complete a Medical Certificate of Cause of Death (here called the death certificate) which has two sections: Part I is the sequential causal pathway resulting in death, and Part II records other conditions the person had pre-mortem that contributed to the death but were not in the causal pathway. Causes may be recorded on any line in Part I or Part II and several causes may be listed on the same line. Commonly the underlying cause of death (UCoD), the condition beginning the causal chain leading to death, is listed on the last line in Part I. The other causes listed above the UCOD in Part I are typically conditions directly leading to death; they are the consequences of the UCoD (e.g., pneumonitis following a fall). Conditions listed in Part II typically describe relevant multi-morbidities. All the conditions listed on the death certificate are coded according to rules for the International Classification of Diseases (ICD) [5]. Then algorithms are used to determine the single underlying cause of death (UCoD) which is used in national statistics. However, the other causes listed on the death certificate can provide important information that is not adequately captured by the UCoD alone. For example, in Australia dementia, including Alzheimer’s disease, as the UCoD rose from being the fourth leading cause of death in 2006 to the second leading cause by 2013 according to the national statistics [6]. During this time the rate of dementia deaths as the UCoD increased by 1.03% per year, however, the rate of deaths with dementia mentioned anywhere else on the death certificate decreased by 0.97% per year [7], so the net effect was that the rate of dementia mentioned anywhere on the death certificate remained stable [8]. This example of dementia illustrates that the UCoD can be influenced by administrative effects such as certification and coding changes. Also, some conditions such as hypertension or congestive heart disease, by their nature, are less likely to be a UCoD and therefore their contribution to death can be understated.

A related issue is the way in which specific causes of death are grouped with other related causes into broader categories. For many practical purposes, the actual numbers or rates of death by cause are less important than their rank order. But this in turn depends on how causes of death are grouped. As Becker et al. note “The rank-order of any causal category depends on the list used… Moreover, a broad cause group, such as ‘all circulatory diseases’, is more likely to score high in the rankings when compared with an individual disease, such as stroke… The process utilized to create condensed tabulations lists should be based on the intended analysis… Any sequence of leading causes is strongly influenced by the criteria according to which the cause-groups of the list are defined” [9].

The main aim of this paper is to present a new method for including both the UCoDs and the other causes recorded on the death certificate into the calculation of national mortality statistics. The objectives of this paper are to: describe a new, data driven, method that uses the patterns of multiple causes of death to calculate the contribution of each cause to national death statistics; compare this method with an alternative method proposed by Piffaretti et al. that allocates arbitrary weights to the UCoD and other contributing causes of death (CCoDs) [10]; and apply both methods using Australian data and compare the effects on rank order of leading causes.

## Methods

### Analysing multiple cause of death data

Piffaretti et al. proposed the following approach for incorporating information from CCoDs into the calculation of the contributions of each cause to the total number of deaths [10]. They only considered the UCoD and CCoDs from Part II of the death certificate (i.e., other Part I conditions are not considered). For each death, weights are assigned to the UCoD and CCoDs in such a manner that the sum of these weights is one [11]. Therefore, the sum of weights of all deaths equals the total number of deaths. For each death, a cause is counted only once; if it is listed as both a UCoD and a CCoD, it is taken as the UCoD and the CCoD is ignored; or if the cause is listed more than once as a CCoD it is counted only once. Let *w*_*ci*_ denote the weight assigned to cause *c* for death *i*. If there are no CCoDs for death *i*, then

$$w_{ci}=\left\{\begin{array}{lc}1&\;\mathrm{if}\;c\;\mathrm{is}\;\mathrm{the}\;\mathrm{UCoD}\\0&\mathrm{otherwise}\end{array}\right.$$

If there are *n*_*i*_ CCoDs for death* i* then

$$w_{ci}=\left\{\begin{array}{cc}p&\mathrm{if}\;c\;\mathrm{is}\;\mathrm{the}\;\mathrm{UCoD}\\\frac{\left(1-p\right)}{n_i}&\mathrm{if}\;c\;\mathrm{is}\;\mathrm a\;\mathrm{CCoD}\end{array}\right.$$

where *p* is an arbitrary weight with 0 ≤ *p* ≤ 1. Then the contribution of cause *c* to all deaths is given by

$$\sum_{i}{w}_{ci}.$$

If *p* = 1 then only the UCoDs are counted, i.e., this is the current way of reporting national death statistics. If *p* = 0 the UCoDs would be completely ignored. Piffaretti et al. illustrated the method using *p* = ½ [10]. Moreno- Betancur et al. used *p* = ¾ and ½ and suggested another method giving equal weights for the UCoD and each of the CCoDs [12]. However, these methods all involve the choice of an arbitrary value, *p*.

To overcome the subjectivity of choosing the value *p* we propose a new data driven method for calculating weights that takes into account the associations that occur between UCoDs and particular CCoDs. For example, ischaemic heart disease as a UCoD commonly has diabetes as a CCoD but is less likely to have lung cancer as a CCoD. In the data driven method the contribution of diabetes in a death with ischaemic heart disease as the UCoD is given more weight than lung cancer. This is due to the common co-occurrence of ischaemic heart disease and diabetes reflecting the causal pathway between them, compared to the less common and less direct link between ischaemic heart disease and lung cancer. In contrast, in methods using arbitrary weights the contributions of these CCoDs would be equal.

The first step is to use all the data to calculate the numbers

$$x_{uc}=\left\{\begin{array}{cc}\frac{N_{c\vert u}}{N_u}&\mathrm{if}\;u\neq c\\0&\mathrm{if}\;u=c\end{array}\right.$$

where *N*_*c|u*_ is the number of deaths with *c* as a CCoD and *u* as the UCoD, and *N*_*u*_ is the total number of deaths with *u* as the UCoD.

The next step is to calculate weights *w*_*ci*_ for each death. Suppose death *i* has *u* as the UCoD and *n*_*i*_ CCoDs. The weight *w*_*ci*_ is defined as

$$w_{ci}=\left\{\begin{array}{cl}{}^{{x}_{uc}}\!\left/ \!{}_{{n}_{i}}\right.,&\text{if one of the CCoDs is} \,c\\1-\sum_{all\;CCoDs}{}^{{x}_{uc}}\!\left/ \!{}_{{n}_{i}}\right.,&\text{if the UCoD is c (i.e.,}\,c=u\text{)}\\0,&\text{if}\,c\,\text{is not the UCoD or a CCoD}\end{array}\right.$$

where *x*_*uc*_ and *n*_*i*_ are defined above. Then the contribution of cause *c* to all deaths is given by $\sum_{i}{w}_{ci}.$

### Australian cause of death records

In Australia each death is certified by a doctor who completes a Medical Certificate of Cause of Death, or the death is referred to the coroner to investigate the circumstances and causes (currently about 12% of deaths are referred to a coroner) [13]. In either case, the cause of death information is lodged directly with the Registrar of Births, Deaths and Marriages in the relevant State or Territory. ICD codes are assigned to each cause and the UCoD is subsequently identified using a combination of automated and manual coding practices. In 1999 ICD 10 was adopted and since 2013 the Iris system for automated processing has been used [14]. Iris is an automatic system for coding multiple causes of death and selecting the underlying cause of death. The system has been designed to accommodate language-dependent aspects of cause of death recording and to improve international comparability. Iris is based on the international death certificate form recommended by WHO and causes of death coded according to ICD-10.

We obtained unit record data from the Australia Coordinating Registry which manages the data from all eight States and Territories. The data were provided in the order recorded on the death certificate (called the entity axis) and as a list with the UCoD recorded first followed by all other causes in alphabetical order (called the record axis). For this paper we used the UCoD from the record axis. In the context of multimorbidity we used the entity axis to identify all the CCoDs which we defined as any Part I causes listed to the right of, or below, the UCoD together with all causes in Part II. Causes listed in Part I above the UCoD were ignored as these should not be part of the pre-mortem pattern of multimorbidity.

The data used for this paper were for all deaths in Australia from 2006 to 2018 inclusive. The starting date of 2006 was chosen because there were major changes which led to a marked discontinuity between 2005 and 2006 [15]. The methods then remained unchanged, except for a change in software in 2013 which did not affect allocations within broad categories of causes [16]. Finally, the most recent data available when this work commenced were for 2018 (when the data were also unaffected by COVID-19). We used records for all people aged 60 years or more and examined the effects of sex and age (three groups: 60 to 74 years, 75 to 84 years, and 85 years and over).

### Categories of causes of death

Various criteria for defining lists of conditions for the analysis of multimorbidity have been published [9, 17, 18]. The main points are as follows.

1. Relevance or fitness for purpose. For this paper the purpose is to analyse data on multiple causes of death among people aged 60 or more in Australia – although the list is likely to be applicable to other countries where most deaths occur from non-communicable diseases in older people, and multiple causes of death are collected and coded.
2. Measurement. In this case all causes of death were coded according to the ICD 10. In Australia ICD 10 codes are assigned to conditions reported on the death certificate or coroner’s findings through a combination of automated and manual coding [16].
3. Prevalence. Common causes should be in singular categories.
4. Categories should be mutually exclusive and exhaustive so that each cause belongs to exactly one category.

The list of 50 leading UCoDs published regularly by the Australian Bureau of Statistics, based on the recommendations of Becker et al*.* [9], formed the foundation for the list developed for this paper. Causes that were uncommon for the study age group were combined with other causes in the same or another chapter in ICD 10 (e.g., infectious diseases, A codes, were grouped with parasitic diseases, B codes, and others). Common causes that were closely related and can sometimes be interchanged were grouped in the same categories (e.g., Alzheimer’s disease, vascular dementia and other dementias, that is G30 and F00-F03, were grouped together even though they are in different chapters). Causes of particular relevance to public health (e.g., suicide, ICD 10 codes X60-X84, Y87.0) were not grouped with other causes. Among the codes for injuries, those that describe the mechanisms of external injuries and would be coded as UCoDs (V, W20-W99, X00-X59, X85-X99, Y00-Y86, Y87.1-Y87.9, Y88-Y99) were grouped with the nature of the injuries which would be coded as CCoDs (i.e., S and T codes). Although codes in the ICD chapter for ‘Symptoms, signs, ill-defined conditions’ would not generally be considered as UCoDs, in the data set used for this paper, terms like ‘senility’ were used sufficiently often as the only cause of death to justify a separate category. This categorisation resulted in 40 categories. See the Appendix for details. The last category, ICD-10 codes U and Z, comprises conditions that are not valid UCoDs but are occasionally used for CCoDs and is included in the list for completeness. For this paper the word ‘cause’, in UCoD or CCoD, refers to the relevant category of causes in the 40-category list.

## Results

### Illustrative example

A simple artificial example of 10 deaths with four causes A, B, C and D, shown in Section I of Table 1, is used to illustrate the calculations of the contributions to the total deaths attributed to each cause. Section II shows the two steps involved in the new method: first using all the data to calculate the number of CCoDs associated with each UCoD (or zero if the CCoD is the same as the UCoD) divided by the frequency of the UCoD; and secondly, for each record separately calculating the weight attributed to each cause. The values from step 2 are added for each cause (column) across all records (rows) to give the total contribution of that cause (shown in the second row of Section IV). Section III shows the alternative method using, for each record, arbitrary weights of *p* = ½ for the UCoD and (1 – *p*) = ½ distributed across all CCoDs, or 1 if there are no CCoDs. Section IV shows the comparison of the method which only considers the UCoD (equivalent to the arbitrary method with *p* = 1), the new data-driven method, and the method using an arbitrary value of *p* = ½. The results are quite similar except for causes C and D. Cause C more often occurs as a CCoD and so makes a bigger contribution to causes of death when either the new method or the method involving arbitrary weights is used, and similarly cause D which is less commonly a CCoD makes a smaller contribution.

**Table 1:** Simple example of ten hypothetical death records with four possible causes of death labelled A, B, C and D. The example shows the calculation of weights using the data driven and arbitrary methods, and the comparison of the methods

***I: Ten hypothetical death records***							
Record ID			UCoD			CCoDs	
1			A				
2			A			A	
3			A			B	
4			A			B C	
5			A			A C	
6			B			A D	
7			B			C D	
8			C			A C A	
9			D			B C	
10			D			A B C	
***II: Data driven weights***							
Step 1: Calculate of the number of CCoDs associated with each UCoD, across all 10 records divided by the frequency of the UCoD, but this is zero if the CCoD is the same as the UCoD							
UCoD	Frequency of UCoD		CCoD				
			A	B		C	D
A	5		0/5	2/5		2/5	0/5
B	2		1/2	0/2		1/2	2/2
C	1		1/1	0/1		0/1	0/1
D	2		1/2	2/2		2/2	0/2
Step 2: Calculation of data driven weights for each cause For each CCoD that is different from the UCoD, weight = (number from Step 1)/(number of CCoDs in record) (that are not the same as the UCoD) For UCoD, weight = 1 – sum of weights for CCoDs							
Record ID	UCoD	CCoDs	# of CCoDs	Data driven weights for each cause			
				**A**	**B**	**C**	**D**
1	A		0	1	0	0	0
2	A	A	0	1	0	0	0
3	A	B	1	1-(2/5) = 3/5	(2/5)/1 = 2/5	0	0
4	A	B C	2	1-(1/5)-(1/5) = 3/5	(2/5)/2 = 1/5	(2/5)/2 = 1/5	0
5	A	A C	1	1-(2/5) = 3/5	0	(2/5)/1 = 2/5	0
6	B	A D	2	(1/2)/2 = 1/4	1-(1/4)-(1/2) = 1/4	0	(2/2)/2 = 1/2
7	B	C D	2	0	1-(1/4)-(1/2) = 1/4	(1/2)/2 = 1/4	(2/2)/2 = 1/2
8	C	A C A	1	(1/1)/1 = 1	0	1–1 = 0	0
9	D	B C	2	0	(2/2)/2 = 1/2	(2/2)/2 = 1/2	1-(1/2)-(1/2) = 0
10	D	A B C	3	(1/2)/3 = 1/6	(2/2)/3 = 1/3	(2/2)/3 = 1/3	1-(1/6)-(1/3)-(1/3) = 1/6
***III. Arbitrary weights,*** if UCoD has weight *p* = 1/2 and there is equal allocation across all unique CCoDs							
Record ID	UCoD		CCoDs	Arbitrary weights for each cause of death			
				A	B	C	D
1	A			1	0	0	0
2	A		A	1	0	0	0
3	A		B	1/2	1/2	0	0
4	A		B C	1/2	1/4	1/4	0
5	A		A C	1/2	0	1/2	0
6	B		A D	1/4	1/2	0	1/4
7	B		C D	0	1/2	1/4	1/4
8	C		A C A	1/2	0	1/2	0
9	D		B C	0	1/4	1/4	1/2
10	D		A B C	1/6	1/6	1/6	1/2
***IV: Comparison of contributions of causes of death***							
			A	B		C	D
UCoD only			50.00%	20.00%		10.00%	20.00%
Data driven weights			52.17%	19.33%		16.83%	11.67%
Arbitrary weights, with *p* = *1/2*			44.17%	21.67%		19.17%	15.00%

### Australian cause of death data

A practical application of these methods is illustrated using Australian cause of death data. The distribution and numbers of causes of death for the 1,663,234 deaths by sex and age group are shown in Table 2. The numbers of deaths increased with age more among women than men. The first quartile, median and third quartiles for the number of CCoDs per death certificate were 0, 1 and 2 respectively for all sex-age groups but the mean numbers increased with age.

**Table 2:** Distribution of deaths in Australia 2006–2018 for all people aged 60 years or more, by sex and age groups and the number of deaths and mean number of contributing causes per death, in brackets

	Age groups (years)			Total
	60–74	75–84	≥ 85	
Men	244,366 (1.06)	293,703 (1.31)	284,125 (1.37)	822,194 (1.26)
Women	152,062 (0.98)	239,729 (1.24)	449,249 (1.28)	841,040 (1.21)
Total	396,428 (1.03)	533,432 (1.28)	733,374 (1.31)	1,663,234 (1.23)

Table 3 shows the effects of various weights allocated to the UCoDs and CCoDs for all deaths among people aged 60 years or more, for the data-driven and arbitrary methods. As might be expected some causes, such as cancers, were much more likely to be listed as UCoDs than CCoDs, so the percentage of all deaths associated with those causes was similar for the method based on UCoDs alone and for the data-driven method but decreased as the arbitrary weight *p* varied from 1 to 0. In contrast, for others such as diabetes and hypertensive disease, the percentage of deaths associated with the cause increased as more weight was given to CCoDs than the UCoD. The proportion of deaths associated with some causes, such as chronic lower respiratory disease, other respiratory disease and other digestive diseases, were similar for all methods and were scarcely affected by variations in the arbitrary weights.

**Table 3:** Numbers and percentages of deaths in Australia 2006–2018 for all people aged 60 years or more by cause: first column is the list of causes of death; the second column is the numbers of deaths with this as the underlying cause; the third column is the percentages of deaths based on data driven method; the remaining columns are percentages of deaths using arbitrary weights varying from 1 (i.e., underlying cause only) to zero (i.e., contributing causes only)

Cause	Number of UCoDs	Data driven	Arbitrary weights varying *p* from 1 to 0						
			**1 = UCoD**	**0.9**	**0.7**	**0.5**	**0.3**	**0.1**	**0 = CCoDs**
Infectious, parasitic disease	37,765	2.17	2.27	2.24	2.18	2.12	2.06	1.99	1.96
Colorectal cancer	58,531	3.43	3.52	3.24	2.67	2.11	1.55	0.98	0.70
Liver cancer	15,821	0.90	0.95	0.86	0.69	0.51	0.34	0.16	0.07
Pancreatic cancer	28,538	1.68	1.72	1.55	1.23	0.90	0.57	0.25	0.09
Lung, tracheal cancer	90,891	5.22	5.46	4.96	3.96	2.96	1.96	0.96	0.46
Melanoma, malignant skin cancer	20,819	1.22	1.25	1.16	0.98	0.80	0.61	0.43	0.34
Breast cancer	26,505	1.56	1.59	1.49	1.29	1.09	0.89	0.69	0.59
Prostate cancer	39,841	2.33	2.40	2.26	1.98	1.70	1.42	1.14	1.00
Lymph, blood cancer	54,034	3.17	3.25	3.06	2.67	2.29	1.91	1.52	1.33
Other malignant neoplasm	142,881	8.63	8.59	8.23	7.52	6.80	6.09	5.38	5.02
Benign neoplasm, blood, metabolic disease	27,882	1.72	1.68	1.81	2.09	2.36	2.63	2.90	3.04
Diabetes	42,902	2.74	2.58	2.86	3.44	4.01	4.58	5.15	5.43
Other endocrine disease	2,247	0.13	0.14	0.19	0.29	0.39	0.49	0.59	0.64
Dementia, Alzheimer's disease	135,475	8.44	8.15	8.07	7.90	7.74	7.58	7.42	7.34
Other mental disorder	6,527	0.56	0.39	0.64	1.14	1.64	2.14	2.63	2.88
Parkinson's disease	18,817	1.09	1.13	1.11	1.08	1.05	1.01	0.98	0.96
Other neurological condition	22,607	1.35	1.36	1.36	1.35	1.34	1.34	1.33	1.33
Eye, ear disease	119	0.01	0.01	0.03	0.09	0.14	0.20	0.25	0.28
Hypertensive disease	18,717	2.05	1.13	1.82	3.21	4.61	6.00	7.39	8.09
Ischaemic heart disease	249,077	14.37	14.98	14.46	13.44	12.41	11.39	10.36	9.85
Cardiac arrhythmia	23,447	1.57	1.41	1.68	2.22	2.77	3.31	3.85	4.12
Heart failure	41,859	2.69	2.52	2.72	3.13	3.53	3.94	4.34	4.54
Cerebrovascular disease	135,272	7.50	8.13	7.75	6.99	6.23	5.46	4.70	4.32
Other circulatory disease	71,395	4.45	4.29	4.52	4.96	5.41	5.85	6.30	6.52
Influenza, pneumonia	33,424	1.95	2.01	1.91	1.72	1.53	1.34	1.15	1.06
Chronic lower respiratory disease	85,154	5.09	5.12	5.12	5.12	5.12	5.13	5.13	5.13
Other respiratory disease	37,332	2.18	2.24	2.28	2.37	2.45	2.53	2.61	2.65
Liver disease	13,177	0.78	0.79	0.81	0.86	0.90	0.94	0.98	1.01
Other digestive disease	44,368	2.54	2.67	2.65	2.60	2.56	2.51	2.47	2.45
Skin disease	5,543	0.31	0.33	0.35	0.39	0.43	0.47	0.51	0.53
Musculoskeletal disease	14,585	0.92	0.88	1.06	1.44	1.81	2.19	2.56	2.75
Kidney disease	47,559	3.00	2.86	3.14	3.72	4.29	4.86	5.43	5.71
Other genitourinary disease	1,014	0.06	0.06	0.07	0.08	0.08	0.09	0.10	0.11
Reproductive, maternal condition	318	0.02	0.02	0.02	0.02	0.02	0.02	0.03	0.03
Perinatal condition, congenital malformation	1,661	0.10	0.10	0.10	0.10	0.10	0.10	0.10	0.10
Symptoms, signs, ill-defined condition	11,109	0.87	0.67	1.00	1.67	2.33	3.00	3.67	4.00
External cause mechanisms, traumatic injuries	24,212	1.50	1.46	1.65	2.03	2.41	2.78	3.16	3.35
Accidental falls	24,761	1.39	1.49	1.36	1.10	0.84	0.58	0.32	0.19
Intentional self-harm	7,048	0.36	0.42	0.38	0.30	0.22	0.14	0.06	0.02
Total	1,663,234	100.00	100.00	100.00	100.00	100.00	100.00	100.00	100.00

**Fig. 1:**
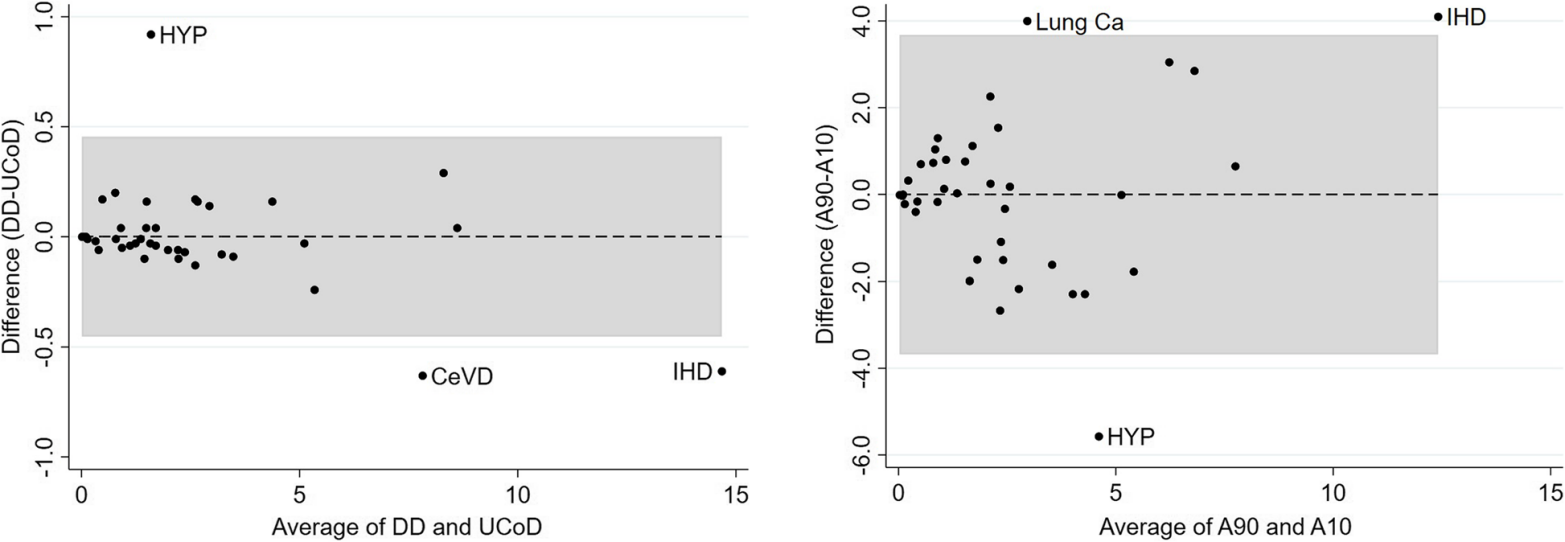
Bland Altman plots comparing percentages of deaths associated with each cause: left panel, data driven estimates (DD) vs underlying cause of death (UCoD), right panel, arbitrary weights with *p* = 0.9 vs *p* = 0.1 (A90 and A10). Points outside shaded areas or limits of agreement, indicate the differences that are beyond what might be expected by chance; these are for hypertensive disease (HYP), cerebrovascular disease (CeVD), ischaemic heart disease (IHD) and lung cancer (Lung Ca). Note the scales for the two graphs are different

Figure 1 shows Bland Altman plots illustrating differences between percentage of deaths associated with different causes when different weighting schemes are used. The left panel shows differences between percentages derived using the data driven approach and the UCoDs. In this case the greatest differences are for hypertensive heart disease, ischaemic heart disease and cerebrovascular disease with hypertensive heart disease given more weight and the other two causes less weight when CCoDs are taken into account. The right panel shows the differences in percentages of deaths for each cause calculated with the arbitrary weights *p* = 0.9 (A90) and *p* = 0.1 (A10). These are greater than the differences shown in the left panel, and so the scales on the vertical axes differ. In the right panel three causes have particularly greater differences between arbitrary weight with *p* = 0.9 (A90, i.e., most weight on the UCoD) and *p* = 0.1 (A10, i.e., most weight on CCoDs) – these are lung cancer which, if present, is mainly recorded as the UCoD, hypertensive disease which is much more commonly reported as a CCoD and ischaemic heart disease which is most commonly reported as a UCoD.

If the causes are ranked in order of decreasing percentage of deaths, the rank order for the most common causes differs by age and between men and women (Tables 4 and 5); for example, the position for dementia rises with age. Within each sex-age group about 6–8 causes are consistently in the top 10 regardless of how the percentages of deaths are calculated, and rank orders among these top causes are affected by small differences in percentages. There is greater similarity in ranks between the UCoDs and the data driven estimates than when the CCoDs are given more weight using the arbitrary method with *p* = 0.5, for example.

**Table 4:** Leading causes of death in Australia 2006–2018 among men aged 60–74, 75–84 and 85 years or more, ranked by using the current method (underlying cause of death alone, UCoD), the new data driven method (DD) and the method using arbitrary weight with *p* = 0.5

Cause	Men aged 60–74			Men aged 75–84			Men aged 85 and over		
	UCoD	DD	*p* = 0.5	UCoD	DD	*p* = 0.5	UCoD	DD	*p* = 0.5
Ischaemic heart disease	1	2	1	1	1	1	1	1	1
Other malignant neoplasm	2	1	2	2	2	2	4	4	8
Lung, tracheal cancer	3	3	3	3	3	9			
Cerebrovascular disease	7	9	8	4	4	6	3	3	3
Dementia, Alzheimer's disease				7	6	4	2	2	2
Chronic lower respiratory disease	5	5	4	5	5	3	5	5	6
Prostate cancer	9	8		6	7	10	6	6	10
Other circulatory disease	8	7	5	8	8	5	7	7	4
Colorectal cancer	4	4		10	10				
Lymph, blood cancer	6	6		9	9				
Kidney disease						8	8	8	5
Diabetes		10	6			7			
Other respiratory disease							10	10	
Heart failure							9	9	7
Hypertensive disease			7						9
Pancreatic cancer	10								
External cause, traumatic injuries			9						
Other mental disorder			10

**Table 5:** Leading causes of death in Australia 2006–2018 among women aged 60–74, 75–84 and 85 years or more, ranked by using the current method (underlying cause of death alone, UCoD), the new data driven method (DD) and the method using arbitrary weight with *p* = 0.5

Cause	Women aged 60–74			Women aged 75–84			Women aged 85 and over		
	UCoD	DD	*p* = 0.5	UCoD	DD	*p* = 0.5	UCoD	DD	*p* = 0.5
Ischaemic heart disease	4	4	2	1	1	1	1	1	2
Dementia, Alzheimer's disease				4	3	2	2	2	1
Cerebrovascular disease	7	7	8	3	4	4	3	3	3
Other malignant neoplasm	1	1	1	2	2	3	5	5	
Chronic lower respiratory disease	5	5	3	5	5	5	7	8	10
Other circulatory disease	9	9	5	7	7	6	4	4	5
Lung, tracheal cancer	2	2	4	6	6				
Colorectal cancer	6	6		8	8				
Breast cancer	3	3	6	10	10				
Other digestive disease							8	10	
Kidney disease			10			9	9	7	7
Heart failure						10	6	6	6
Lymph, blood cancer	8	8		9	9				
Influenza, pneumonia							10		
Hypertensive disease			9			7		9	4
Pancreatic cancer	10	10							
Diabetes			7			8			
Cardiac arrhythmia									8
Symptoms, signs ill-defined conditions									9

## Discussion

In this paper we have described a new method for including multiple causes of death in statistics that summarise the contribution for each cause to the total number of deaths in a population. The method takes account of the patterns of associations among UCoDs and CCoDs. It is driven by the data and does not involve an arbitrary choice of weights. We illustrated the method using a simple artificial example which demonstrates how the frequencies with which CCoDs occur with specific UCoDs affect their contributions to the results. To demonstrate the practical application of the method we applied it to Australian mortality data for people aged 60 years or more. Compared to the usual method based only on UCoDs, with the new method the percentage of deaths attributed to a condition like hypertensive disease (which is a common CCoD) almost doubles from 1.13% to 2.05%, and the percentages are attributed to the related conditions of ischaemic heart disease and cerebrovascular disease decrease. Similarly, the percentage contributions attributable to diabetes, dementia (including Alzheimer’s disease) and heart failure are all higher using the new method. For some conditions, like chronic obstructive pulmonary disease, and other respiratory conditions, the percentage of deaths varies little with different calculation methods because they occur about equally commonly as UCoDs or CCoDs. There are some causes, notably cancers, which are usually recorded as the UCoD with few, if any CCoDs. For these causes the percentages are only slightly lower based on the new method compared with the usual, UCoD only method, but can be considerably reduced if arbitrary weights are used. This is an important outcome achieved by the data driven method compared to that achieved using arbitrary weights. Cancers are legitimately the underlying cause of these deaths and reducing the ‘burden’ associated with these cancer deaths and attributing it to other causes would be difficult to justify from a public health perspective. Thus, reducing the relative importance of cancer does not make sense in the same way that reducing the rank order of ischaemic heart disease in deference to hypertensive diseases or diabetes does.

Several authors have suggested alternative methods for addressing the growing concern that, as the prevalence of multi-morbidity is increasing due to population ageing, the exclusive use of UCoDs in national mortality statistics does not adequately represent the importance of some conditions in terms of the population health burden [10–12]. The methods that have been proposed involve arbitrary choices of weight to be assigned to UCoDs and CCoDs without regard to the patterns that occur among these causes. The new data driven method is designed to overcome this limitation by taking into account the joint frequencies of conditions. The results in Table 3 show how this approach can increase or decrease the contributions of different conditions according to these patterns.

In this paper the unit of analysis is the death and not the cause of death. Thus, in common with other authors, each death is counted once so every death has the same weight in the total count, regardless of the number of CCoDs mentioned on the death certificate [3, 10, 12]. This differs from an analysis in which the total number of times a cause is mentioned in all death certificates may be the statistic of interest. In this case a death with numerous CCoDs will be more influential than one with only the UCoD reported. Counting each death just once is comparable with the current practice of using UCoDs only and it is more robust to differences in coding practices, for example, between countries which differ in the number of CCoDs commonly reported [3]. However, the data driven method uses the relative frequency of each cause across all deaths with the same UCoD in the term *x*_*uc*_ which is used to calculate the weights *w*_*ci*_. But, in the data driven method the influence for each cause is affected by the number *n*_*i*_ of CCoDs on death certificate *i* (a feature shared with methods using arbitrary weights).

When causes of death are listed in rank order, as in Tables 4 and 5, the striking feature is that, within age and sex groups the ranks vary little between the data driven method and the current method based on UCoDs (but this effect is not necessarily found with the arbitrary weights). The reason for this robustness of ranks of causes is the use of groups of closely related causes (e.g., related to the vascular system, or the respiratory system). Most of the ‘exchange’ of weights occurs within these groups, e.g., between dementia as a CCoD when ischaemic heart disease is the UCoD and dementia as the UCoD when ischaemic heart disease as a CCoD. This phenomenon provides insights into the importance of how the groups of causes are defined. The categories used in this paper were based on the recommendations of Becker et al. [9] with modifications used by the Australian Bureau of Statistics, such as grouping Alzheimer’s disease with other dementias. Provided such a list of aetiologically related causes is used, results in this paper show that the percentages of deaths associated with different causes and the rank order of causes are quite robust to inclusion of CCoD information based on patterns within the data. This finding should provide confidence that the standard method, used by the World Health Organisation and many countries, does in fact provide a good representation of the relative importance of cause specific mortality rates.

Nevertheless, the methods discussed in this paper are not appropriate for universal use, for example as the international standard for reporting death statistics. In countries with incomplete registration of death, inadequate identification of causes of death, or where CCoDs are poorly recorded or not recorded at all, trying to take account of multiple causes of death is not sensible or feasible. Complete registration and improving the quality of UCoDs must remain the priority. However, for countries with high quality multiple cause of death data, two forms of statistical tabulations could be routinely reported: the current one based on UCoDs only, and another that uses the multiple cause data. This two-part approach would directly address the concerns that multimorbidity is inadequately represented in national statistics.

There are a number of limitations to the data and methods used in this study. Firstly, death certificates are not always filled in correctly. For example, several causes may be listed on the last line in Part I. In this paper those on the right of the UCoD were taken as CCoDs (i.e., assuming they were contributing causes that should have been in Part II) and those listed above and before the UCoD in Part I were ignored (assuming they were consequences of the UCoD). Other authors have taken the same approach, for example Piffaretti et al. calculated estimates both including and excluding these Part I causes [10]. Secondly, there is substantial evidence that some causes of death, e.g., diabetes [19–21] and dementia [22–25], are poorly recorded on death certificates as UCoDs or CCoDs even when the person is known in their lifetime to have the condition. Sensitivities of the order of 40—50% for diabetes and dementia have been reported, even for these causes listed anywhere on the death certificate [26].

If national statistics are based only on UCoDs the effects of causes which may be considered as risk factors for other causes, in particular endocrine, nutritional and metabolic diseases, may be underestimated [11]. Indeed, Goldberg et al. have suggested that differences in the way such conditions are coded as UCoDs or CCoDs can explain apparent differences in disease patterns between countries [27]. This issue is important for distinguishing between deaths directly attributable to COVID-19 (deaths from COVID-19) and those where COVID-19 was a CCoD (death with COVID-19) [28].

There are several important strengths of this study. In Australia the quality of death certification is high with approximately 88% of death certificates completed by a registered medical practitioner and the remainder obtained from coroners’ reports. Additionally, the data cover a period of thirteen years when there were very few changes in coding practices or the software used for processing the multiple causes listed on the death certificates, and there were no major changes that would impact on population mortality. By adopting the principle that each death is counted only once, we have ensured that the new method is robust to certification variations in the number of CCoDs reported on the death certificate.

## Conclusion

A new method is proposed for calculating the percentages of deaths attributed to different causes when multiple cause of death data are available. It takes into account the patterns that occur between UCoDs and CCoDs as listed on the death certificate. Unlike previously proposed methods it does not rely on arbitrary choices of weights and does not treat all CCoDs equally. The application of the method to Australian mortality data shows how multi-morbidity can affect the percentages of deaths associated with different causes. The new method does not greatly affect the rank order of conditions, confirming the validity of the current practice based UCoDs alone. However, it does produce results that more adequately reflect the contribution of certain causes to overall mortality burden. It would be suitable for use by national statistical agencies to produce mortality tables that reflected the information available on multiple causes to complement the current tables based only on UCoDs.

## Data Availability

The datasets supporting the conclusions of this article are available in the UQeSpace repository in 10.48610/25f6577.

## Appendix

**Table:** **Table 6** Categorisation of ICD-10 codes to groups relevant for men and women aged over 60 years in Australia

Label	Causes of death	ICD 10 codes
1	Infectious, parasitic disease	A00-A45, A47-A99, B00-B07, B08.0, B08.2-B08.3, B08.5-B08.9, B09-B17, B19-B85, B87-B99, D59.3, G00-G06, H65-H67, H70, J00-J06, J20-J22, J85-J86, N29.0, N30, N33.0, N34, N39.0, N74.0-N74.4, O98.0-O98.3, O98.7, P35.0, P37.0, P37.3-P37.4
2	Colorectal cancer	C18-C21, C26.0
3	Liver cancer	C22
4	Pancreatic cancer	C25
5	Lung, tracheal cancer	C33-C34
6	Melanoma, malignant skin cancer	C43-C44
7	Breast cancer	C50
8	Prostate cancer	C61
9	Lymph, blood cancer	C81-C96, D45-D46, D47.0-D47.1, D47.3-D47.5
10	Other malignant neoplasm	C00-C17, C23-C24, C26.1-C26.9, C27-C32, C35-C42, C45-C49, C51-C60, C62-C80, C97-C99
11	Diabetes	E10.0-E10.1, E10.3-E10.9, E11.0-E11.1, E11.3-E11.9, E12.0-E12.1, E12.3-E12.9, E13.0-E13.1, E13.3-E13.9, E14.0-E14.1, E14.3-E14.9, O24.0-O24.2
12	Other endocrine disease	E00-E09, E15-E27, E28.0-E28.1, E28.3-E28.9, E29-E39, E47-E49, E69, E81-E82, E89, E91-E99
13	Dementia, Alzheimer's disease	F00-F03, G30
14	Psychiatric and other mental disorders	F04-F99
15	Parkinson's disease	G20
16	Other neurological condition	G07-G19, G21-G29, G31-G44, G46-G79, G81-G99
17	Eye, ear disease	H00.1-H00.9, H01-H59, H60.2-H60.9, H61-H64, H68-H69, H71-H99
18	Hypertensive disease	I10-I11, I13-I15
19	Ischaemic heart disease	I20-I25
20	Cardiac arrhythmia	I47-I49
21	Heart failure	I50-I51
22	Cerebrovascular disease	I60-I69
23	Other circulatory disease	G45, I00-I09, I16-I19, I26-I46, I52-I59, I70-I84, I86-I99
24	Influenza, pneumonia	J09-J18
25	Chronic lower respiratory disease	J40-J47
26	Other respiratory disease	D86.0, D86.2, D86.9, J07-J08, J19, J23-J33, J34.1-J34.9, J35-J39, J48-J84, J87-J99
27	Liver disease	B18, K70-K77
28	Other digestive disease	I85, K00-K61, K62.0-K62.1, K62.4-K62.9, K63-K69, K78-K99
29	Skin disease	A46, B08.1, B08.4, B86, H00.0, H60.0-H60.1, J34.0, L
30	Musculoskeletal disease	M
31	Kidney disease	E10.2, E11.2, E12.2, E13.2, E14.2, I12, N00-N28, N29.1-N29.9, N31-N32, N33.1-N33.9, N35-N38, N39.1-N39.9, Q61.0-Q61.3
32	Other genitourinary disease	N40-N42, N51-N61, N65-N73, N74.5-N74.9, N78-N79, N84-N99
33	Reproductive, maternal condition	D25, E28.2, K62.2-K62.3, N43-N50, N62-N64, N75-N77, N80-N83, O00-O23, O24.3-O24.9, O25-O97, O98.4-O98.6, O98.8-O98.9, O99
34	Perinatal condition, congenital malformation	G80, P00-P34, P35.1-P35.9, P36, P37.1-P37.2, P37.5-P37.9, P38-P99, Q00-Q60, Q61.4-Q61.9, Q62-Q99, R95
35	Symptoms, signs, ill-defined conditions	R00-R94, R96-R99
36	External cause mechanisms, traumatic injuries	S, T, V, W20-W99, X00-X59, X85-X99, Y00-Y86, Y87.1-Y87.9, Y88-Y99
37	Accidental falls	W00-W19
38	Intentional self-harm	X60-X84, Y87.0
39	Miscellaneous diseases and disorders	D00-D24, D26-D44, D47.2, D47.6-D47.9, D48-D58, D59.0-D59.2, D59.4-D59.9, D60-D85, D86.1, D86.3-D86.8, D87-D99, E40-E46, E50-E68, E70-E80, E83-E88, E90
40	Invalid UCOD	U, Z

## Abbreviations

Ca: Cancer
CCoD: Contributing cause of death
CeVD: Cerebrovascular disease
COVID-19: Coronavirus disease 2019
DD: Data driven estimates
HYP: Hypertensive disease
ICD: International Classification of Diseases
IHD: Ischaemic heart disease
UCoD: Underlying cause of death
WHO: World Health Organization
